# Effect of integrated perioperative rehabilitation intervention under the fast-track surgery concept on stress and complications in patients undergoing craniocerebral injury surgery

**DOI:** 10.3389/fsurg.2022.1014211

**Published:** 2023-01-06

**Authors:** Bin Zhao, Heng Wang

**Affiliations:** ^1^ Department of Taditional Chinese Medicine Rehabilitation, The Third Affiliated Hospital, Hengyang Medical School, University of South China, Hengyang, China; ^2^ Trauma Center, The Third Affiliated Hospital, Hengyang Medical School, University of South China, Hengyang, China

**Keywords:** craniocerebral injury, FTS concept, integrated perioperative rehabilitation intervention, stress response, complications

## Abstract

**Objective:**

To observe the intervention effect of perioperative rehabilitation intervention of integrated medical care the concept of FTS on stress response and postoperative complications in patients undergoing craniocerebral injury surgery.

**Methods:**

70 patients with Traumatic brain injury (TBI) admitted to the Department of Neurosurgery of our Hospital from January 2019 to December 2021 were as the research objects and were divided into general group and FTS group according to the random number table method, with 35 cases in each group. The general group was intervened with perioperative basic nursing measures for TBI, and the FTS group was intervened with perioperative rehabilitation model of integrated medical care under the concept of FTS on the basis of the general group. The two groups of patients were compared in hemodynamic indexes (heart rate, mean arterial pressure), stress hormone levels (CORT, GLU, E), changes in motor neurological function (GCS score, NHISS score, FMA score), occurrence of postoperative complications (infection, pressure sores, rebleeding, central hyperthermia), short-term quality of life (SF-36) before and after the intervention.

**Results:**

After intervention, the levels of HR, MAP, COR, GLU, and E were significantly lower in FTS group than in the general group (all *P* < 0.05). After intervention, the Fugl-Meyer score and Barthel index score of upper and lower extremities in both groups were significantly higher than those before intervention, and the FTS group was higher than the general group, and the difference was statistically significant (*P* < 0.05). After the intervention, the NIHSS scores were significantly lower in both groups than before the intervention, and the FTS group was lower than the general group, and the differences were statistically significant (*P* < 0.05). Short-term physical function, somatic pain, physical function, general health status, social function, energy, mental health, and emotional function scores were significantly higher in the FTS group than in thegeneral group, and all differences were statistically significant (*P* < 0.05). The total incidence of infection, pressure ulcers, rebleeding, central high fever and other complications in the FTS group was significantly lower than that in the general group (*P* < 0.05).

**Conclusion:**

The implementation of integrated perioperative rehabilitation interventions under the concept of FTS for patients with TBI can significantly alleviate patients’ stress, promote recovery, reduce the incidence of complications, and improve short-term quality of life, which is worthy of clinical promotion.

## Preface

Traumatic brain injury (TBI) is a common acute injury in neurosurgery, which is mainly caused by external violent factors acting directly or indirectly on the head, including scalp lacerations, scalp hematomas, intracranial hematomas, skull fractures, concussions and other symptoms (1, 2). At present, there is still a tendency to attach importance to saving lives and neglect functional rehabilitation of patients in the treatment and nursing of TBI, so that many patients, especially the critically ill ones, do not receive early and effective functional rehabilitation nursing exercises in a timely manner, and the incidence of sequelae is high (3). In addition, due to the trauma caused by surgery to the organism, patients are often prone to postoperative complications such as venous thrombosis, which severely affects the psychological and physiological response of patients with craniocerebral trauma and adversely affects their return to their families and society (4, 5). Therefore, how to reduce perioperative physiological and psychological stress reactions in patients with craniocerebral trauma has become a key concern for clinical health care professionals.

Fast-track surgery (FTS) is a clinical practice program that emphasizes the concept of patient-centered services and refers to the optimization of perioperative clinical pathways in order to reduce the patient's stress response to surgery, thereby reducing complications, shortening the length of hospital stay, and promoting patient recovery (6, 7). The core of the concept is to reduce the stress response to surgery and restore organ function, with the aim of promoting patient recovery, not just for early discharge. In recent years, with the progress of surgical techniques, the concept of perioperative rehabilitation interventions for TBI has become more evident. The integration of medical and nursing care refers to the process in which physicians and nurses work together to provide medical care to patients through communication and coordination between medical and nursing care under the premise of equal autonomy and with certain expertise and competence (8, 9). FTS focuses on a multidisciplinary approach to problem solving and decision making through the implementation of an integrated medical and nursing approach, which brings together the strengths of each field and integrates them with a common goal, so that an integrated medical and nursing approach to rapid perioperative rehabilitation interventions is necessary to enable patients to recover after surgery (10, 11).

Domestic and international studies (12, 13) have reported that integrated medical and nursing rehabilitation interventions based on the FTS concept have been used in thoracic surgery, spine surgery, orthopedics, and cancer patients with more extensive results making them safe, effective, and improving their prognosis. However, in China, reports on the application of integrated medical and nursing perioperative rehabilitation interventions under the FTS concept in the perioperative period of TBI are rare. Therefore, it is necessary for us to further explore and improve the integration of medical, nursing and health care for the rapid perioperative rehabilitation program of TBI patients, so as to truly realize the rapid rehabilitation of craniocerebral injury surgery patients and provide better and more effective medical care services for patients.

## Object and method

### Source of study subjects

TBI patients who were admitted to our hospital from January 2019 to December 2021 and planned to undergo surgical treatment were selected strictly according to the inclusion and exclusion criteria. In this study, patients were divided into a general group (i.e., basic perioperative care measures for TBI) and an FTS group (the general group was intervened with an integrated medical and nursing perioperative rehabilitation model based on the FTS concept) using a completely randomized design.

### Inclusion criteria

The presence of patients with a clear history of cranial trauma with a clear mechanism of injury, confirmed by cranial CT examination at the time of admission as traumatic craniocerebral injury; the patients were admitted within 24 h after the injury; the ASA classification was II to III; the patients with stable vital signs; the absence of alcohol and various drug dependence; and the patients and their families signed the informed consent.

### Exclusion criteria

People with consciousness, cognitive disorders and mental diseases; Patients with surgical contraindications; Patients with serious organ dysfunction; Suffering from malignant tumors; Patients with other severe injuries, multiple fractures, or brain stem injuries that were difficult to reverse.

### Interventions

Patients in both groups were given routine emergency treatment for TBI. These included: ① Promptly clear the foreign body of the respiratory tract, avoiding vomit, and tracheal intubation to keep the respiratory tract open if necessary; ② Promptly treat the wound to prevent hemorrhagic shock; ③ Establish two intravenous channels and rehydration to prevent water-electrolyte disorders; ④ Actively give anti-infection treatment according to medical advice, and give protection to patients with open craniocerebral injury.

On the basis of basic emergency treatment, patients in the general group were given basic perioperative care measures for craniocerebral injury, including: ① Pre-operative preparation, which included skin preparation, blood sampling, blood preparation, urinary catheterization and various skin tests to help patients get into surgery as soon as possible and as early as possible; Patients received routine health information instruction given by the neurosurgery nurse before surgery, including risk factors for complications, dietary guidance, precautions for medication and preventive measures. ② After the operation, the patient's vital signs were closely monitored, and the patient was instructed to have a reasonable diet, functional exercise of the limbs in bed and training of daily living skills according to the patient's specific condition.

Patients in the FTS group received the same nursing and rehabilitation instructions as the general group, and also received perioperative rehabilitation intervention based on the concept of FTS.

I. The investigator assisted the chief of neurosurgery and the head nurse to establish the FTS team, and the core members were doctors, nurses (with titles of nurse practitioner and above), anesthesiologists, convalesiologists and dietitians in the department. To jointly develop a comprehensive rehabilitation intervention plan for TBI patients by reviewing relevant literature reports
II. Intervention content, (1) Preoperative preparation: ① Health education in collaboration with medical and nursing: before operation, propaganda manuals were distributed to patients and their families to explain the nursing process in detail, timely grasp the psychological and emotional changes of patients and take active and effective intervention measures, so as to improve the preoperative tension, anxiety and fear of patients. ② Pre-operative condition assessment: nursing staff enhanced communication with the patient, collected information on the patient's general condition, family situation and condition, and the nurse-patient collaborated to assess the condition and develop an individualized care plan, and the attending physician and rehabilitation nutrition physician developed an individualized diet plan according to the patient's specific condition to improve the rapid rehabilitation care process. (2) Intraoperative preparation: ① Optimize anesthetic treatment: anesthetic treatment methods that were less stressful to the organism and help postoperative analgesia should be chosen as much as possible to ensure that the postoperative pain level of patients was in a tolerable state, so as to effectively reduce the stress response of patients. ② Ensure that the patient's body was warm during the operation: care should be taken to ensure that the patient's body was in a warm state during the surgical operation to avoid aggravating the patient's stress reaction by getting cold. (3) Postoperative preparation: ① Cooperative postoperative care: In postoperative diet, complication and pain care, patient trust was obtained through enhanced communication, and individualized care was provided according to the patient's condition to improve patient compliance. ② Dietary care: The patient was conscious after operation, and was given enteral nutrient powder with warm water after head CT showed no obvious abnormalities. The patient was given semi-liquid diet on 1 day after operation, and normal diet on 2 days after operation. ③ Early postoperative rehabilitation exercise: after the patient became conscious after surgery, we assisted him/her to position the limbs correctly, and turned him/her once every 2 h. After the patient's condition became stable, we instructed him/her to perform rehabilitation exercise of various joints, 10 times for each joint, 15 min for each exercise, 2 times/day. At the same time, we instructed him/her to complete rehabilitation training such as turning and sitting, bed bridging, etc., 15 min for each exercise, 2 times/day. Patients were encouraged to get out of bed as soon as possible and were instructed to perform walking training with the help of tools. (4) Continuity of care: We established a WeChat exchange group, regularly pushing health knowledge and home care attention measures, etc., investigating patients’ conditions and giving professional guidance by means of follow-up visits, etc., and at the same time regularly tell the patient to return to the hospital for review.

## Observation indicators

### Hemodynamic indexes, stress hormone levels

The heart rate (HR) and mean arterial pressure (MAP) were recorded before and after the intervention as two time points. At each time point, 2 ml of arterial blood was extracted and centrifuged at 3,000 r/min for 10 min, and the supernatant was extracted after 10 min, and each supernatant was labeled by number and stored in a refrigerator at −70°C. After all specimens were collected, the stress response indexes of patients with TBI during perioperative period were detected by enzyme-linked immunosorbent assay (ELISA).

### Changes in motor neurological function

The Fugl-Meyer Motor Function Scale was used to evaluate the upper limb and lower limb motor function of the two groups before and after interventio, in which the motor function score of the upper extremity ranged from 0 to 66 and the motor function score of the lower extremity ranged from 0 to 34, with higher scores indicated better motor function. The NIHSS scores were used to the neurological changes of the two groups before and after intervention, with scores ranging from 0 to 42, with higher scores indicating more severe neurological impairment. The Barthel index scale was used to evaluate the changes of the activities of daily living of the two groups before and after the intervention, with scores ranging from 0 to 100, the higher the score, the better the patient's ability to perform daily living.

### Short-term quality of life

The quality of life scale (SF-36 scale) was used to assess the quality of life in both groups after the intervention. The scale included 8 dimensions of physical function, somatic pain, physical function, general health status, social function, energy, mental health, and emotional function, and each dimension was scored from 0 to 100, with higher scores indicating better quality of life for patients.

### Occurrence of postoperative complications

Including infection, pressure sores, rebleeding, and central hyperthermia.

### Statistical analysis

SPSS 20.0 software was used to statistically analyze the obtained data, and the measurement data were expressed as (*x* ± *s*), and *t*-test was used for comparison between groups, and paired *t*-test was used for comparison within groups; the count data were expressed as rate (%), and chi-square test was used for comparison. The difference was considered statistically significant at *P* < 0.05.

## Results

### Comparison of general information of patients in two groups

The patients were divided into general group and FTS group by random number table method, and there were 35 cases in each group. All patients were scored GCS immediately after admission, including mild craniocerebral injury: 13–15 points; Moderate craniocerebral injury: 9–12 points; Severe craniocerebral injury: 3–8 points. There was no statistically significant difference between the two groups in age, gender, cause of injury, cultural level, clinical diagnosis, place of residence and degree of injury (*P* > 0.05), which was comparable, see Table 1.

**Table 1:** Comparison of general survey data between the two groups of patients.

Data	General group (*n* = 35)	FTS group (*n* = 35)	*t* or *χ*^2^ value	*P* value
Sex (male, %)		25 (71.43)	22 (62.86)	0.583	0.445
Age (years; mean, SD)		45.87 ± 7.23	46.12 ± 8.84	0.130	0.897
Cause of injury (*n*, %)	Traffic accident	8 (22.86)	7 (20.00)	0.283	0.963
	External force blow	6 (17.14)	6 (17.14)		
	Fall injury	15 (42.86)	17 (48.57)		
	Fall from height	6 (17.14)	5 (14.29)		
Cultural level (*n*, %)	Elementary school and below	5 (14.29)	4 (11.43)	0.524	0.770
	Junior high school	12 (34.29)	10 (28.57)		
	High school and above	18 (51.43)	21 (60.00)		
Clinical diagnosis (*n*, %)	Subarachnoid hemorrhage	4 (11.43)	6 (17.14)	0.688	0.876
	Cerebral contusion	18 (51.43)	16 (45.71)		
	Epidural hematoma	5 (14.29)	4 (11.43)		
	Subdural hematoma	8 (22.86)	9 (25.71)		
Place of residence (*n*, %)	Rural	10 (28.57)	12 (34.29)	1.063	0.588
	Suburb	13 (37.14)	9 (25.71)		
	Urban	12 (34.29)	14 (40.00)		
Degree of injury (*n*, %)	Light	4 (11.43)	3 (8.57)	0.291	0.865
	Moderate	14 (40.00)	13 (37.14)		
	Heavy	17 (48.57)	19 (54.29)		

### Comparison of hemodynamic indexes and stress hormone levels between the two groups before and after the intervention

**Figure 1:**
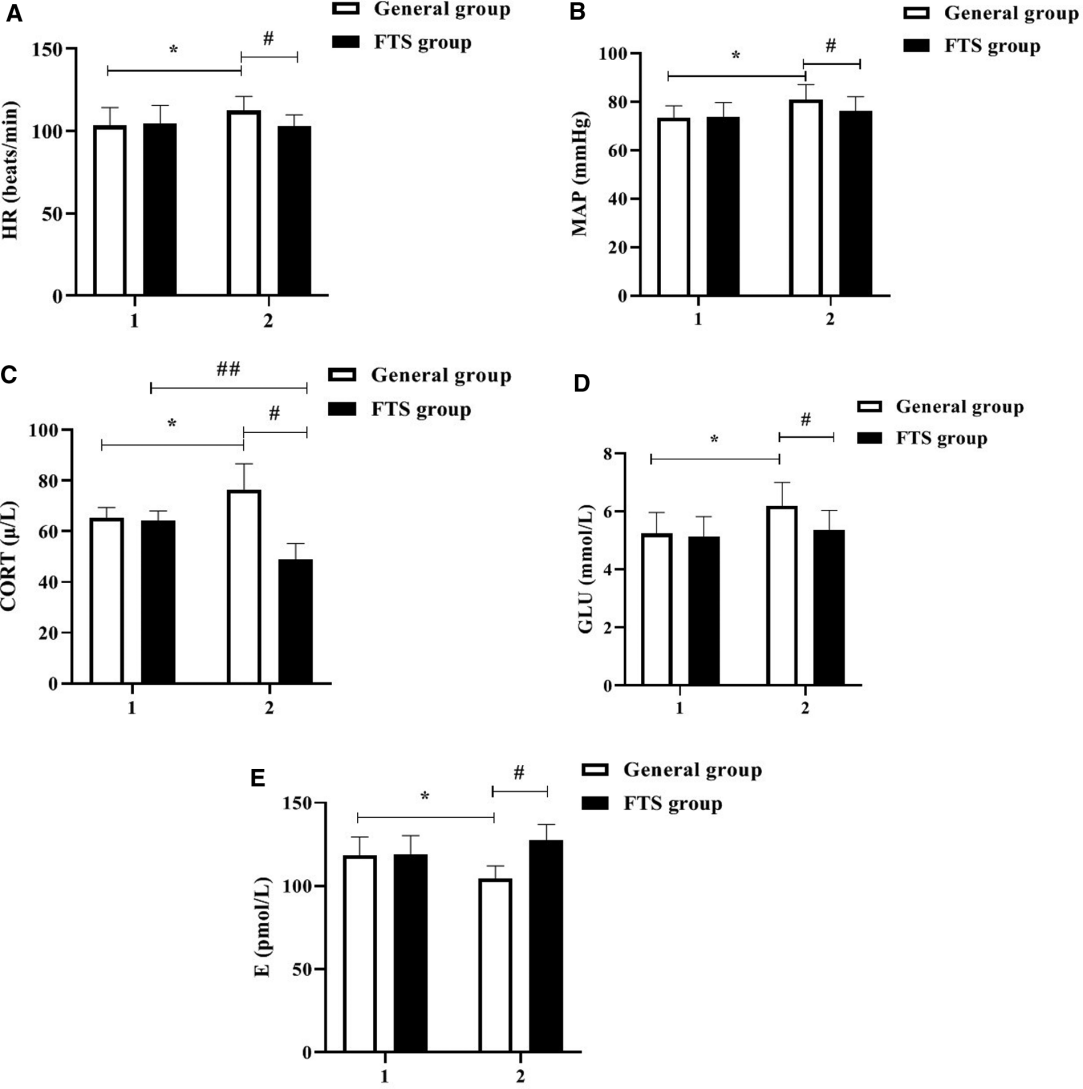
Comparison of hemodynamic indexes and stress hormone levels between the two groups before and after the intervention. In the figure, 1 represents before intervention and 2 represents after intervention. * Indicates the comparison of the general group before and after intervention, *P* < 0.05; # Indicates the comparison of the FTS group with the general group after intervention, *P* < 0.05; ## Indicates the comparison of the FTS group before and after intervention, *P* < 0.05. Figure (**A-E**) shows the comparison of HR, MAP, COR, GLU, and E levels in the two groups, respectively.

There was no statistically significant difference in the levels of HR, MAP, COR, GLU and E between the two groups before the intervention (*P* > 0.05); after the intervention, the levels of HR, MAP, COR, GLU and E in the FTS group were significantly lower than those in the general group (all *P* < 0.05). See Figure 1.

### Comparison of motor nerve function between the two groups before and after intervention

**Figure 2:**
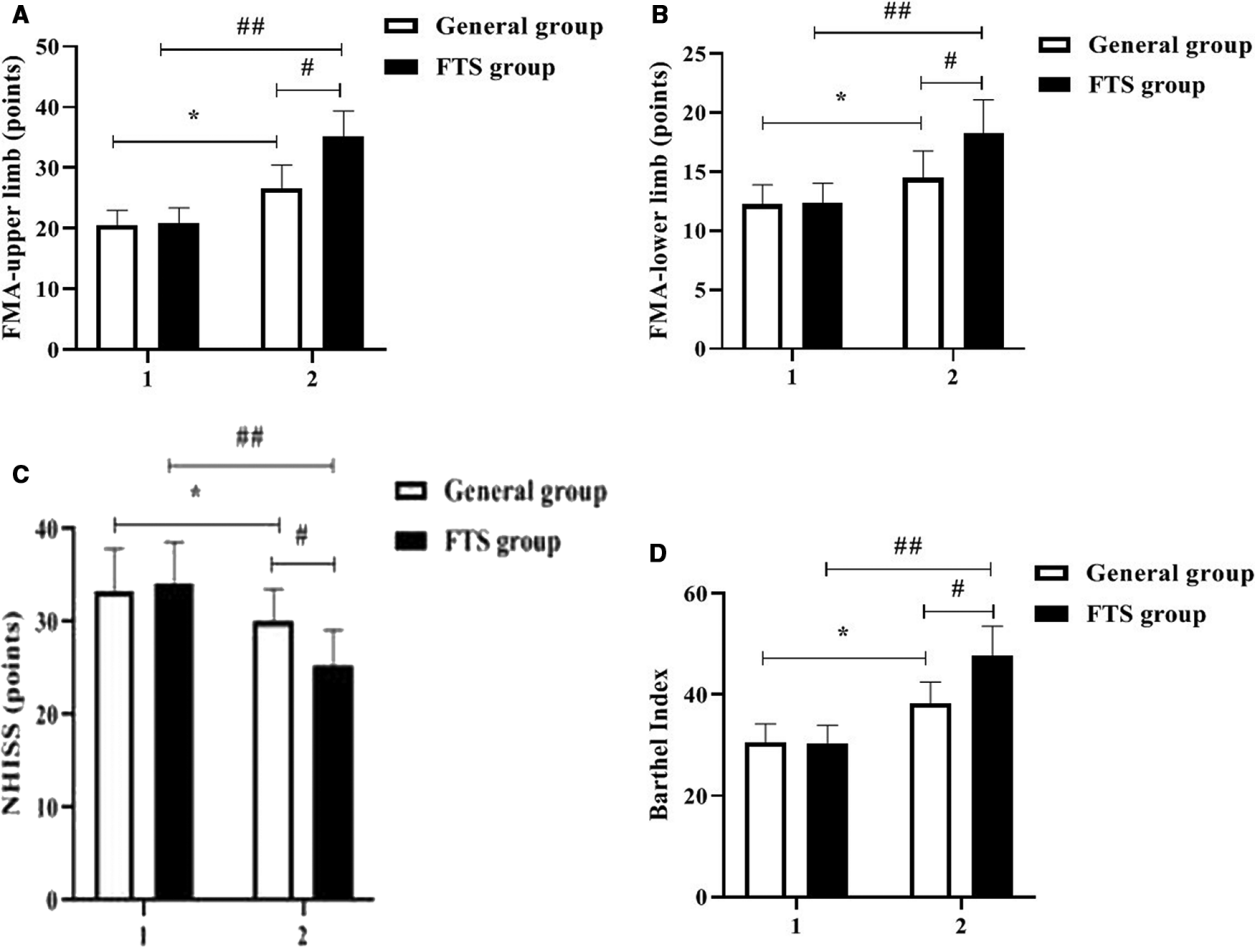
Comparison of motor nerve function between the two groups before and after intervention. In the figure, 1 represents before intervention and 2 represents after intervention. * Indicates the comparison of the general group before and after intervention, *P* < 0.05; # Indicates the comparison of the FTS group with the general group after intervention, *P* < 0.05; ## Indicates the comparison of the FTS group before and after intervention, *P* < 0.05. Figure (**A-D**) shows the comparison of FMA-upper limb, FMA-lower limb, NIHSS score, and Barthel index between the two groups, respectively.

Before the intervention, there were no significant differences in FMA score, Barthel index and NIHSS score of upper and lower limbs between the two groups (*P* > 0.05); After intervention, the Fugl-Meyer score and Barthel index score of the upper and lower limbs of the two groups were significantly higher than those before intervention, and the FTS group was higher than that of the general group (*P* < 0.05). After the intervention, the NIHSS scores were significantly lower in both groups than before the intervention, and the FTS group was lower than the general group, and the differences were all statistically significant (*P* < 0.05). See Figure 2.

### Comparison of short-term quality of life between the two groups

**Figure 3:**
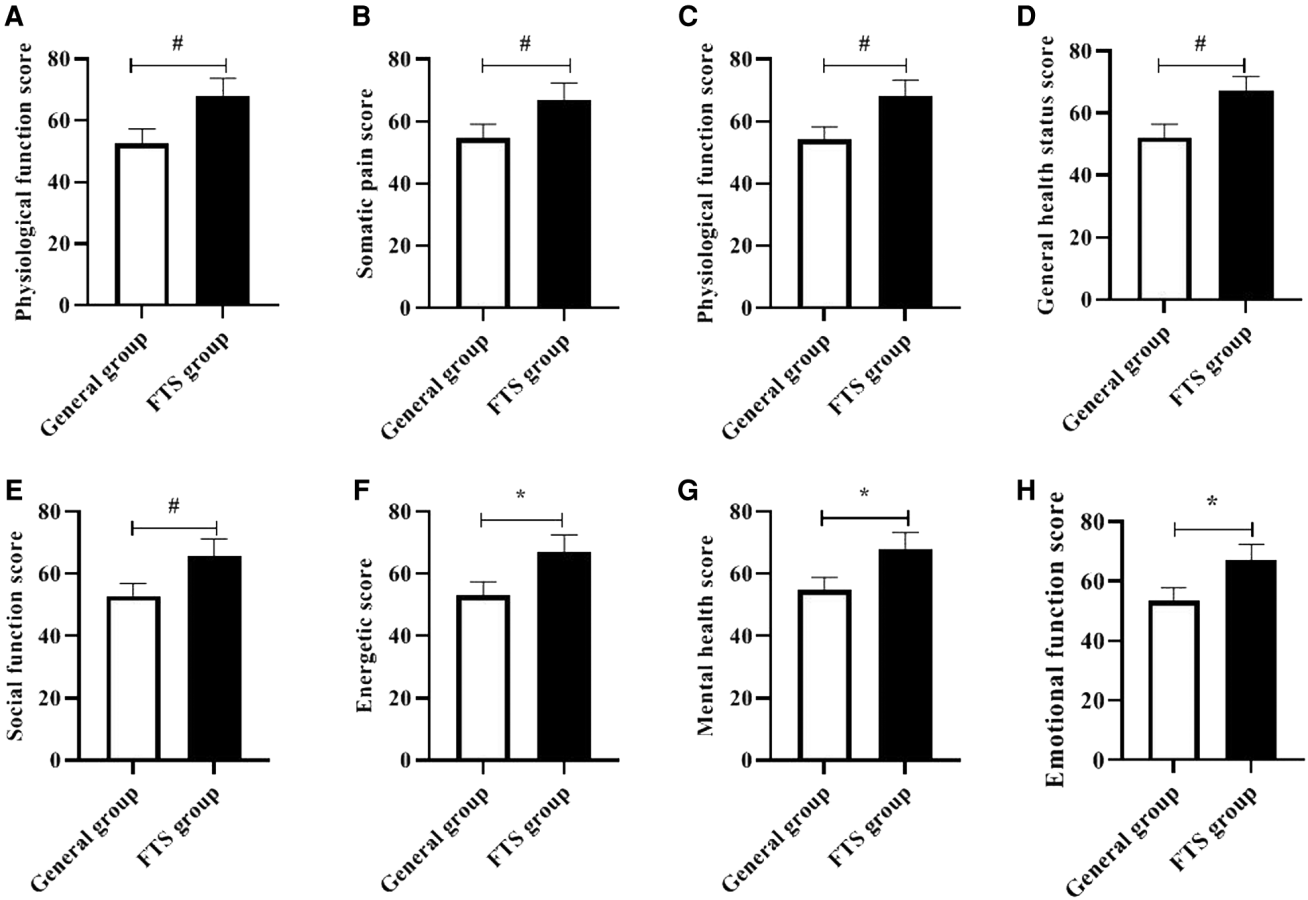
Comparison of short-term quality of life between the two groups. #,* Indicates *P* < 0.05 compared with the general group. Figure (**A-H**) shows the comparison of physical function, somatic pain, physiological function, general health status, social function, energy, mental health, and emotional function scores between the two groups, respectively.

The short-term physical function, somatic pain, physical function, general health status, social function, energy, mental health, and emotional function scores of the FTS group were significantly higher than those of the general group, and all differences were statistically significant (*P* < 0.05). See Figure 3.

### Comparison of the occurrence of postoperative complications in the two groups

This investigation found that the postoperative complications in the conventional group and the FTS group were mainly concentrated in infection, pressure ulcers, rebleeding, and central hyperthermia. A comparison of the occurrence of complications between the two groups showed that the total complication rate in the FTS group was significantly lower than that in the general group, and the difference was statistically significant (*P* < 0.05). See Table 2.

**Table 2:** Comparison of the occurrence of postoperative complications between the two groups of patients (*n*, %).

Group	Infection	Pressure sores	Re- bleeding	Central hyperthermia	Total
General group (*n* = 35)	7 (20.00)	4 (11.43)	3 (8.57)	5 (14.29)	19 (54.29)
FTS group (*n* = 35)	3 (8.57)	2 (5.71)	2 (5.71)	2 (5.71)	9 (25.70)
*χ*^2^ value	-	-	-	-	5.117
*P* value	-	-	-	-	0.024

## Discussion

TBI is a common surgical multidisease, and studies (14) show that craniocerebral trauma accounts for 15%–20% of systemic injuries in China, second only to extremity injuries. Patients with TBI can progress to hemiparesis with dilated pupils and varying degrees of headache, vomiting, optic papillary edema, and impairment of consciousness, thinking, sensation, and movement, and their condition progresses rapidly and is not easily controlled, which can easily pose a serious threat to their life safety if not treated promptly from time to time (15, 16). Surgery is now the basic method of treatment of TBI, but surgery as a strong stimulation factor, can make the body produces a series of physiological and psychological reaction, appear different degree of tension, anxiety, depression, hypertension, myocardial ischemia induced, immunosuppression, reduced pain threshold and other adverse reactions, seriously affect the patient's rehabilitation therapy (17, 18). Therefore, it is important to adopt effective nursing interventions to reduce perioperative patient stress, improve treatment outcomes and enhance quality of life.

FTS breaks the traditional concept and advocates preoperative health education, shortening the time of fasting water, multi-mode analgesia, intraoperative heat preservation, early postoperative water intake, early activity and other measures, which involve a variety of disciplines (19). Medical and nursing integration is a process in which doctors and nurses work together to provide quality medical and nursing care to patients through communication and coordination between doctors and nurses under the premise of equal freedom, with significant clinical application (20). In this study, the integrated perioperative rehabilitation intervention under the FTS concept was applied to the clinical care of patients with TBI. The results showed that after the intervention, the levels of HR, MAP, CORT, GLU, and E were significantly better in the postoperative FTS group than in the general group, indicating that the integrated perioperative rehabilitation intervention under the FTS concept was effective in relieving patients’ stress reactions. Studies (21, 22) have shown that E and CORT are sensitive indicators of the body's stress response, and that integrated perioperative rehabilitation interventions under the FTS concept have a certain stress-relieving effect, with psychological care, dietary care, and exercise care taking into account the physiological, psychological, and social needs of patients, relieving their physiological and psychological stress responses and keeping them in an optimal state of comfort. After intervention, the FMA and Barthel index scores of the upper and lower limbs of the two groups were significantly higher than those before intervention, and the FTS group was higher than that of the general group. The NIHSS scores of the two groups were significantly lower than those before intervention, and the FTS group was lower than that of the general group (all *P* < 0.05). The results suggested that after FTS concept intervention, the recovery effects of motor function, daily living ability and neurological function in the FTS group were significantly better than those in the general group. Related studies (23, 24) concluded that the FTS concept can effectively promote the rapid postoperative recovery of neurosurgical patients, which includes a number of measures such as preoperative preparations, surgical operation methods, optimization of anesthesia methods, postoperative rehabilitation exercises and regular follow-up visits after discharge, so as to systematically and scientifically guide patient care and promote the rapid recovery of patients’ postoperative conditions. Zhong et al.'s study (25) showed that under the guidance of the FTS concept, scientific and reasonable dietary care, exercise care and psychological care, strengthening their exercise capacity through passive limb training and other exercise instructions, and strengthening skin care can effectively prevent the occurrence of complications such as venous thrombosis and pressure sores, thus reducing patients’ perioperative stress and improving their compliance with treatment and care and postoperative quality of life. In this study, the short-term quality of life of the FTS group was better than that of the conventional group, and the incidence of postoperative complications was lower than that of the conventional group, indicating that the integrated perioperative rehabilitation intervention under the FTS concept can effectively reduce the incidence of postoperative complications and improve the short-term postoperative quality of life of patients, supporting the above findings.

In conclusion, the implementation of integrated perioperative rehabilitation interventions under the concept of FTS for patients with TBI can significantly alleviate patients’ stress, promote recovery, reduce the incidence of complications, and improve short-term quality of life, which is worthy of clinical promotion. However, there are still shortcomings in this study, such as the small sample size, and further expansion of the sample size is needed for related studies.

## Data Availability

The original contributions presented in the study are included in the article/Supplementary Material, further inquiries can be directed to the corresponding author/s.
